# Tissue-specific Network Analysis of Genetic Variants Associated with Coronary Artery Disease

**DOI:** 10.1038/s41598-018-29904-7

**Published:** 2018-07-31

**Authors:** Xiao Miao, Xinlin Chen, Zhijun Xie, Honghuang Lin

**Affiliations:** 1 Innovation Research Institute of Traditional Chinese Medicine, Shanghai University of Traditional Chinese Medicine, Shanghai, China; 2 Department of Cardiovascular Medicine, Longhua Hospital, Shanghai University of Traditional Chinese Medicine, Shanghai, China; 3 College of Basic Medical Science, Zhejiang Chinese Medical University, Hangzhou, Zhejiang Province China; 4 Section of Computational Biomedicine, Department of Medicine, Boston University School of Medicine, Boston, MA USA

## Abstract

Coronary artery disease (CAD) is a leading cause of death worldwide. Recent genome-wide association studies have identified more than one hundred susceptibility loci associated with CAD. However, the underlying mechanism of these genetic loci to CAD susceptibility is still largely unknown. We performed a tissue-specific network analysis of CAD using the summary statistics from one of the largest genome-wide association studies. Variant-level associations were summarized into gene-level associations, and a CAD-related interaction network was built using experimentally validated gene interactions and gene coexpression in coronary artery. The network contained 102 genes, of which 53 were significantly associated with CAD. Pathway enrichment analysis revealed that many genes in the network were involved in the regulation of peripheral arteries. In summary, we performed a tissue-specific network analysis and found abnormalities in the peripheral arteries might be an important pathway underlying the pathogenesis of CAD. Future functional characterization might further validate our findings and identify potential therapeutic targets for CAD.

## Introduction

Coronary artery disease (CAD) is a leading cause of death worldwide^1–3^. CAD is heritable, and a familial history of CAD is associated with a significantly increased CAD risk^4^. Recent genome-wide association studies (GWAS) have identified more than one hundred genetic loci associated with CAD^5–11^. However, most of these loci are located outside of protein coding regions, and their implication on CAD is still largely unknown. Moreover, many studies have been focused on the association of single variants with CAD. Given that the majority of complex diseases including CAD are caused by the interplay of many genetic and environmental factors^12,13^, it is important to jointly investigate the combinatory effects of multiple genetic variants on biological pathways and interaction networks^14–16^.

The gene function is highly dependent on the tissue where the gene is expressed^17^, which is controlled by very distinct regulatory programs^18^. Genes with tissue-specific expression have shown important physiological processes for complex organisms^19^. However, functional studies of human genes have been traditionally carried out on specific cell lines, and the characterization of tissue-specific interactions is predominantly based on a small sample size. Recent advances in next generation sequencing provide an unprecedented opportunity to profile gene expression in a much larger scale of human samples^20,21^. An excellent example is the Genotype-Tissue Expression (GTEx) project, which has characterized the association of genetic variants with gene expression in nearly 50 different types of human tissues that were collected from approximately 1000 individuals^22^.

The objective of our current study is to understand the function of CAD-related genetic loci through the gene interaction network. The associations of individual variants with CAD were summarized into gene-level associations, which were then combined with experimentally validated gene interactions as well as gene coexpression in coronary artery. A tissue-specific interaction network then built to examine the interactions between CAD-related genes and their potential functions in terms of biological pathways.

## Results

### Gene-level Association with CAD

**Figure 1:**
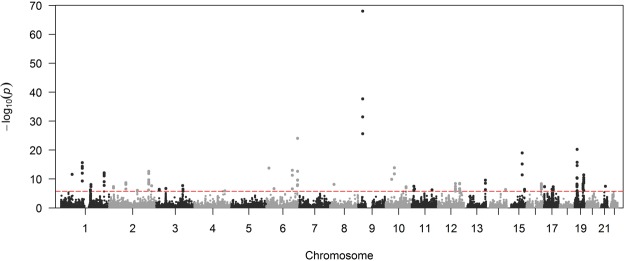
Manhattan plot of gene-level association with CAD. Each dot represents one gene. The x-axis represents chromosome positions, whereas the y-axis represents the log_10_(*P*). The red dash indicates *P* < 0.05/26228 = 1.91 × 10^−6^. Only autosomal variants were included in the analysis.

The association of genetic variants with CAD was previously investigated^7^. The study included 9,455,778 common variants, among which 2,213 variants passed the genome-wide significance (*P* < 5 × 10^−8^). Variant-level associations were then summarized into gene-level associations using fastBAT^23^. Variants within 50 kb of the gene region were collapsed and jointly modelled. Figure 1 is the Manhattan plot showing the association of each of the 26,228 genes with CAD. The Q-Q plot of the associations is shown in Supplementary Figure 1. A total of 143 genes were significantly associated with CAD after Bonferroni correction of multiple testing (*P* < 0.05/26,228 = 1.91 × 10^−6^). Table 1 shows the top 25 genes associated with CAD, and the full list of CAD-related genes is shown in Supplementary Table 1. The most significant gene was *CDKN2B-AS1* (*P* = 9.45 × 10^−69^), which is the antisense of *CDKN2B* that is located at the 9p21 locus. The locus has long been recognized to be associated with various cardiovascular diseases^24–28^. We also performed a sensitivity analysis by expanding the flanking sequence to 100 kb to include more regulatory variants. As shown in Supplementary Figure 2, the association was highly correlated (R^2^ = 0.88).

**Table 1:** Top 25 genes associated with CAD.

Chromosome	Gene	#Variants within the gene	Gene association with CAD (*P* value)	Best variant with the gene	Best variant association with CAD (*P* value)
9p21.3	*CDKN2B-AS1*	283	9.45E-69	rs2891168	2.29E-98
9p21.3	*CDKN2B*	96	2.04E-38	rs7028268	4.98E-48
9p21.3	*CDKN2A*	119	3.42E-32	rs3217992	1.03E-42
9p21.3	*CDKN2A-AS1*	104	2.26E-26	rs3217992	1.03E-42
6q25.3	*LPA*	180	8.34E-25	rs55730499	5.39E-39
19p13.2	*SMARCA4*	219	5.88E-21	rs56289821	4.44E-15
15q25.1	*ADAMTS7*	216	9.37E-20	rs4468572	4.44E-16
19p13.2	*LDLR*	256	1.71E-16	rs56289821	4.44E-15
1p13.3	*SORT1*	114	2.18E-16	rs7528419	1.97E-23
15q25.1	*MORF4L1*	134	6.37E-16	rs4468572	4.44E-16
19p13.2	*MIR6886*	182	2.13E-15	rs56289821	4.44E-15
1p13.3	*MYBPHL*	113	3.96E-15	rs7528419	1.97E-23
10q11.21	*C10orf142*	135	1.31E-14	rs1746050	6.28E-13
6p24.1	*PHACTR1*	656	1.59E-14	rs9349379	1.81E-42
1p13.3	*PSRC1*	128	1.88E-14	rs7528419	1.97E-23
6q23.2	*LINC01312*	113	9.18E-14	rs12202017	1.98E-11
2q33.2	*WDR12*	51	2.08E-13	rs115396314	5.11E-18
6q26	*PLG*	184	2.28E-13	rs2315065	2.88E-34
1q41	*MIA3*	99	7.04E-13	rs67180937	1.01E-12
1q41	*TAF1A-AS1*	111	9.66E-13	rs35700460	1.38E-12
1p13.3	*CELSR2*	159	9.70E-13	rs7528419	1.97E-23
2q33.2	*CARF*	67	1.28E-12	rs115654617	3.12E-18
10q11.21	*LINC00841*	222	1.57E-12	rs1870634	5.55E-15
1q41	*TAF1A*	132	1.96E-12	rs35700460	1.38E-12
1p32.2	*PLPP3*	211	2.40E-12	rs9970807	5.00E-14

### Tissue-specific Interaction Network Related to CAD

**Figure 2:**
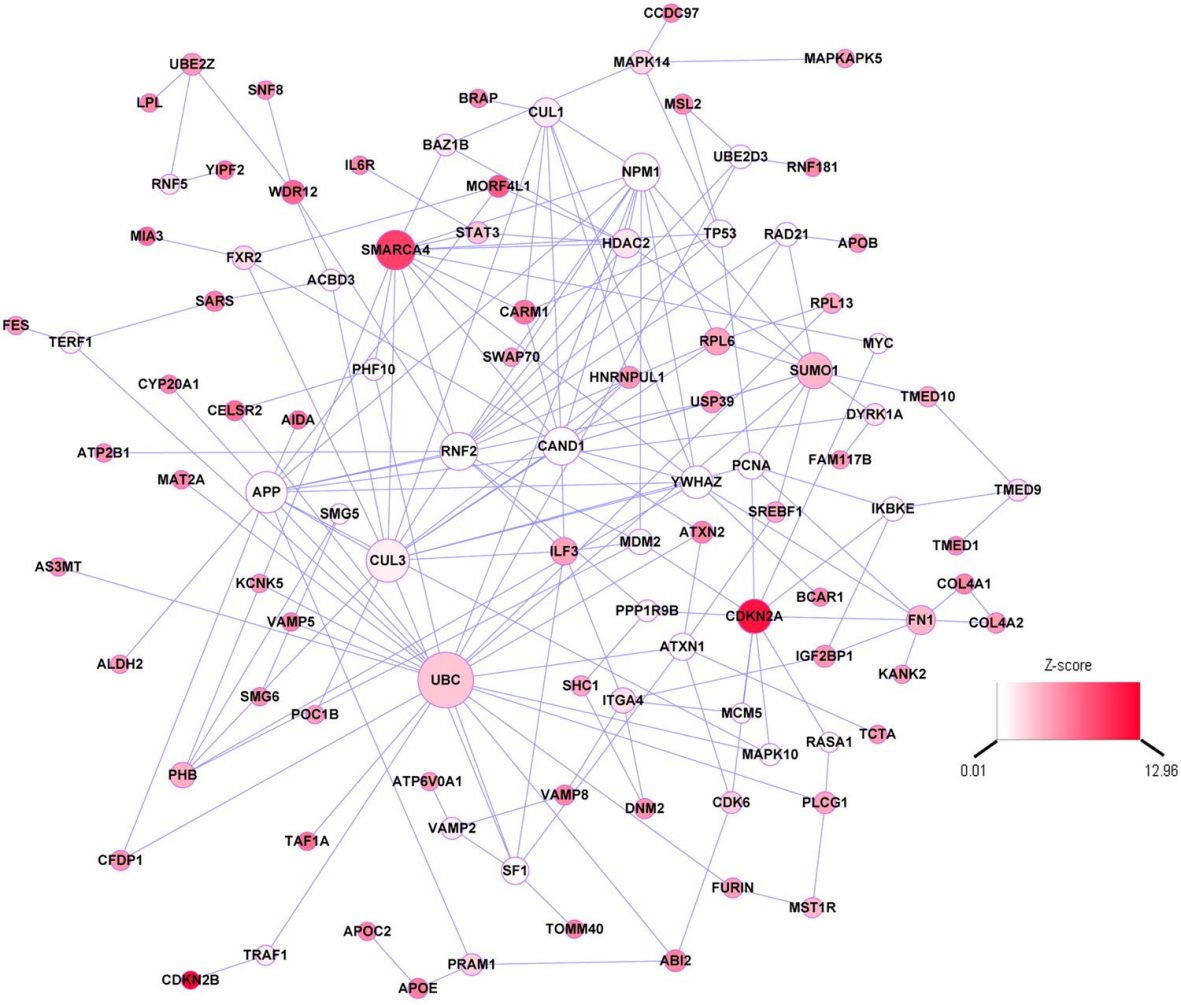
CAD-related network derived from protein-protein interaction. Each node represents one gene, wheras each edge represents the interaction between two genes. The nodes were colored to represent their association with CAD: red color represents genes that were associated with CAD, white color represents genes were not associated with CAD. The node size is proportional to the number of edges that the node is connectted to.

We then built a CAD-related interaction network by integrating GWAS and gene coexpression in coronary artery. As shown in Fig. 2, the network is comprised of 102 nodes and 182 edges. Each node represents one gene, and each edge represents the interaction between two genes. These genes were interconnected together, so not all of the 143 CAD-related gene were included. Among the 102 genes in the network, 53 were CAD-related genes (*P* < 1.91 × 10^−6^).

We then examined the potential functional implication of the network using WebGestalt^29^. As shown in Table 2, six disease pathways were significantly enriched with genes in the network (FDR <0.1). The most significant pathway is abnormalities of the peripheral arteries (*P* = 1.63 × 10^−6^, FDR = 3.52 × 10^−3^), which includes eight CAD-related genes. In addition, many CAD-related genes were involved in xanthomatosis (*P* = 2.06 × 10^−5^, FDR = 2.96 × 10^−2^) and cerebral ischemia (*P* = 4.94 × 10^−5^, FDR = 5.33 × 10^−2^).

**Table 2:** Most significant disease pathways enriched with CAD-related genes (FDR < 0.1).

Disease pathway	#Genes in the pathway	Ratio of enrichment	*P* value	FDR	Overlapping Genes
Abnormalities of the peripheral arteries	97	8.98	1.63E-06	3.52E-03	*COL4A1; COL4A2; APOB; APOE; APP; LPL; TP53; BAZ1B*
Xanthomatosis	19	22.93	2.06E-05	2.96E-02	*APOB; APOC2; APOE; LPL*
Cerebral ischemia	46	11.84	4.94E-05	5.33E-02	*COL4A1; COL4A2; APP; TP53; BAZ1B*
Coronary artery disease	51	10.68	8.20E-05	6.80E-02	*APOB; APOE; LPL; TP53; BAZ1B*
Abnormality of the coronary arteries	53	10.28	9.89E-05	6.80E-02	*APOB; APOE; LPL; TP53; BAZ1B*
Neoplasm of the adrenal cortex	11	29.71	0.00011	6.80E-02	*CDKN2B; MDM2; TP53*

A recent study^30^ of CAD prioritized 184 candidate genes as the most likely causal genes for CAD based on functional evidence. These genes were involved in 286 modules. Interestingly, 26 of those candidate genes were also included in our network (enrichment *P* < 2.2e-16). We also examined if genes in the network are potential drug targets. DrugBank^31^ was queried, and 13 genes in the CAD-related network were reported as therapeutic targets for at least one known or developing drug, suggesting the potential of network analysis to identify drug targets.

### Identification of Key Drivers in the Network

We also examined the structure of the CAD-related network, and tested if they were any key drivers of the network. Each gene in the network was evaluated by the association of its neighbors with CAD, and the distribution was then compared with random distribution. A single gene, *UBC*, turned out to be one of the most important genes in the network. The gene was nominally associated with CAD (*P* = 1.25 × 10^−3^) but did not reach the genome-wide significance cutoff (*P* < 1.91 × 10^−6^). However, it interacted with 22 other genes, including 10 CAD-related genes, suggesting that it might be an important regulator of the CAD-related pathways.

We also calculated the weighted centroid of the network, which took into consideration of both the weight of neighboring genes as well as the strength of interactions (represented by the absolute value of the correlation coefficient between two genes). As shown in Supplementary Table 2, *UBC*, together with *CAND1* and *SMARCA4*, were among the top centroid genes.

## Discussion

One challenge in the post-GWAS era is to understand molecular mechanisms underlying the association of GWAS loci with diseases. Given that many genes are only expressed in certain tissues, it is important to study gene functions in a tissue-specific manner. Here we built a CAD-related interaction network in coronary artery, and found that many of genes in the network were involved in abnormalities of the peripheral arteries. Our study suggests that the disturbing of peripheral artery functions might be an important pathway leading to CAD.

*UBC* was found to be a key driver of the network. The gene encodes ubiquitin C, a highly conserved gene^32^ that acts as a potential target of N-Formylmethionine^33^. In combination with proteasome, the ubiquitin-proteasome system (UPS) is responsible for the degradation of up to 80–90% of proteins in mammalian cells^34^, which is essential to the removal of non-functional or damaged polypeptides^35^. The ubiquitination also regulates multiple cardiac signal transduction pathways^36–38^ as well as the promotion of pathologic hypertrophic growth of cardiomyocytes^39,40^.

It is interesting to see that *APOB* and *APOE* were both involved in multiple pathways enriched with CAD-related genes. *APOB* encodes apolipoprotein B, a lipoprotein that is known to be involved in the development of CAD^41,42^. The inclusion of apolipoprotein B level into risk models would significantly improve the prediction performance of future risk of coronary heart disease^43^. Genetic variations in *APOB* were also associated with Mendelian diseases such as familial hypobetalipoproteinemia^44^. *APOE* encodes a ligand that binds to both low density lipoprotein receptor and APOE-specific receptor. It is involved in the regulation of cholesterol and the metabolism of lipoproteins, and the polymorphisms of *APOE* are associated with atherosclerosis^45^ and coronary heart disease^46,47^.

Tissue-specific expression has been recognized as an important pattern for many diseases. Traditional expression quantitative trait loci (eQTLs) studies were mostly focused on gene expression in the blood due to convenience and accessibility. However, these studies have limited power to detect tissue-specific expression profiles. Recent efforts are being devoted to identify eQTLs across a variety of tissue types^22,48,49^, which would empower an in-depth study of gene function among different types of tissues^50,51^.

GWAS provided an unbiased approach to screen a huge amount of genetic variants with complex diseases. In order to correct for multiple testing and reduce false positives, a stringent significance cutoff is applied (typically *P* < 5 × 10^−8^), which on the other hand, could result in the loss of many true associations. By integrating gene interactions, we were able to reprioritize gene signals, and identify some additional associations that might be otherwise missed because of moderate significance. One example is *TGFB1*, which encodes a transforming growth factor, which plays an important role in the regulation vascular smooth muscle^52^. The gene was significantly associated with CAD after summarizing genetic variants within the gene region (*P* = 6.26 × 10^−9^). However, the most significant SNP within the gene region was rs15052 (*P* < 2.21 × 10^−7^), which did not reach the genome-wide significance cutoff (*P* < 5 × 10^−8^). A recent study^10^ that combined UK Biobank together with CARDIoGRAMplusC4D 1000 Genomes-based GWAS and the Myocardial Infarction Genetics and CARDIoGRAM Exome found that rs8108632 within the *TGFB1* region was significantly associated with CAD (*P* = 4.04 × 10^−8^). Our results suggest that a combination of joint testing of multiple variants together with gene interactions could offer additional power to identify novel susceptibility loci for complex diseases.

Given that many top variants identified by GWAS are just tagging variants, it is challenging to pinpoint the casual variants/genes. For example, the most significant variant associated with body mass index is rs9930506, which is located within the intron of *FTO*. However, it was found that the causative gene at the locus is actually *IRX3*, which is located ~480 kb away from the top SNP rs9930506^53^. Appropriately 6.22% of *cis*-eQTLs in coronary artery are even more than 500 kb away from the corresponding genes^22^, suggesting that causal genetic variants could be far away from the genes. On the other hand, the inclusion of long-range regulation could also result in an increase of irrelevant genes. Therefore it is useful to include additional annotations such as those derived from the ENCODE Project^54^ and the Roadmap Epigenomics Project^55^.

Our study has several limitations. The interaction network was built from previously reported protein interactions. However, it was estimated that only a small proportion of interactions have been characterized^56–58^. Therefore, many important interactions might still be missing and thus were not included in the current analysis. In addition, tissue-specific interactions were estimated from the correlation of gene expression, which had limited power to identify interactions between genes with low expression. It is also worth to note that the key driver analysis is a hypothesis generating approach solely based on the strength of associations of individual genes and the network structure. It is by no means to indicate causality between genes. Further experimental validation is needed to understand the mechanism of associations and the biological processes involved in the etiology of CAD.

In conclusion, we performed a tissue-specific network analysis of genetic variants associated with CAD. Our study underscored the role of abnormalities in the peripheral arteries in the pathogenesis of CAD. Future functional characterization of CAD-related gene might identify potential therapeutic targets for CAD.

## Methods

### Association of Genetic Variants with CAD

The summary statistics of genetic variants associated with CAD was obtained from the CARDIoGRAMplusC4D 1000 Genomes-based genome-wide association study (http://www.cardiogramplusc4d.org/data-downloads/), which included 48 studies with a total of 60,801 CAD cases and 123,504 controls^7^. The raw genotypes were imputed to 1000 Genomes phase 1 v3 that included more than 38 million variants, but only common variants (minor allele frequency ≥1%) were included in the current analysis.

### Derivation of Gene-level Association with CAD

Variant-level associations were summarized into gene-level associations using fastBAT^23^. For each gene, we took into account of all the variants within 50 kb of the gene region, and joint tested their association with CAD. Given that tens of thousands of genes were tested, a nominal significance cutoff (*P* < 0.05) could result in a number of false positives. Therefore Bonferroni correction was used, which is a conservative adjustment that assumes all tests are independent. Genes with p-value less than 0.05/N were considered as significant, where N was number of tested genes.

### Construction of a Tissue-specific Interaction Network

Gene interactions were obtained from the iRefIndex database^59^. Self-interactions or interactions involved in non-human genes were removed from downstream analyses. Gene coexpression was based on 173 coronary artery samples from the GTEx project^22^. Pearson correlation was calculated for each interaction gene pair, and pairs with normalized correlation higher than 0.25 were considered as coexpression in the tissue.

### Construction of Gene Interaction Network Related to CAD

A dense module searching strategy^60^ was implemented to identify modules enriched with CAD-related genes. Each gene was assigned a score equivalent to the absolute value of the z-score of the association with CAD. Seed genes were defined as those significantly associated with CAD after Bonferroni correction. The module searching started with a single seed gene. Neighboring genes were then added sequentially to the module if the addition would increase the overall module score^61^, which was defined as ${Z}_{m}=\frac{\sum {g}_{i}}{\sqrt{k}}$, where k was the number of genes in the module, and ${g}_{i}$ was the score of gene i. The process iterated until no more genes could be added. Modules derived from different seed genes were highly overlapped and thus were merged to create a combined interaction network.

### Identification of Key Drivers in the Network

Key drivers were defined as genes that interact with more CAD-related genes than what would be expected from a randomly selected gene set with an equal number of genes. These genes are pivotal to the structure of the network and might be potential targets for further functional characterization. The score of each gene was defined as the z-score of the association with CAD. The Kolmogorov–Smirnov test was then used to assess the deviation of scores of neighboring genes from the random expectation. To correct for multiple testing, Bonferroni adjustment was used and genes with *P* < 0.05/N were defined as key drivers, where N was the total number of genes in the network. In addition, we also calculated the weighted centroid of the network, which was defined as ${C}_{i}={g}_{i}+\sum _{k}|{w}_{i,k}|\ast {g}_{k}$, where *C*_*i*_ was the weighted centroid of gene i, and *w*_*i*,*k*_ was the correlation coefficient between gene i and gene k, and *g*_*k*_ is the score of gene k.

## Electronic supplementary material

Supplementary materials
