# Supplementary material for: Tissue-specific Network Analysis of Genetic Variants Associated with Coronary Artery Disease
Source: Sci Rep. 2018 Jul 31;8:11492. doi: 10.1038/s41598-018-29904-7 (PMC6068195; doi:10.1038/s41598-018-29904-7)
Supplement: Supplementary file 1 — Supplementary materials [file 41598_2018_29904_MOESM1_ESM.docx]

**Tissue-specific Network Analysis of Genetic Variants Associated with Coronary Artery Disease**

Xiao Miao, MD PhD; Xinlin Chen, MD; Zhijun Xie, PhD; Honghuang Lin, PhD

**Supplementary Figure 1. Q-Q plot of gene-level associations with CAD**


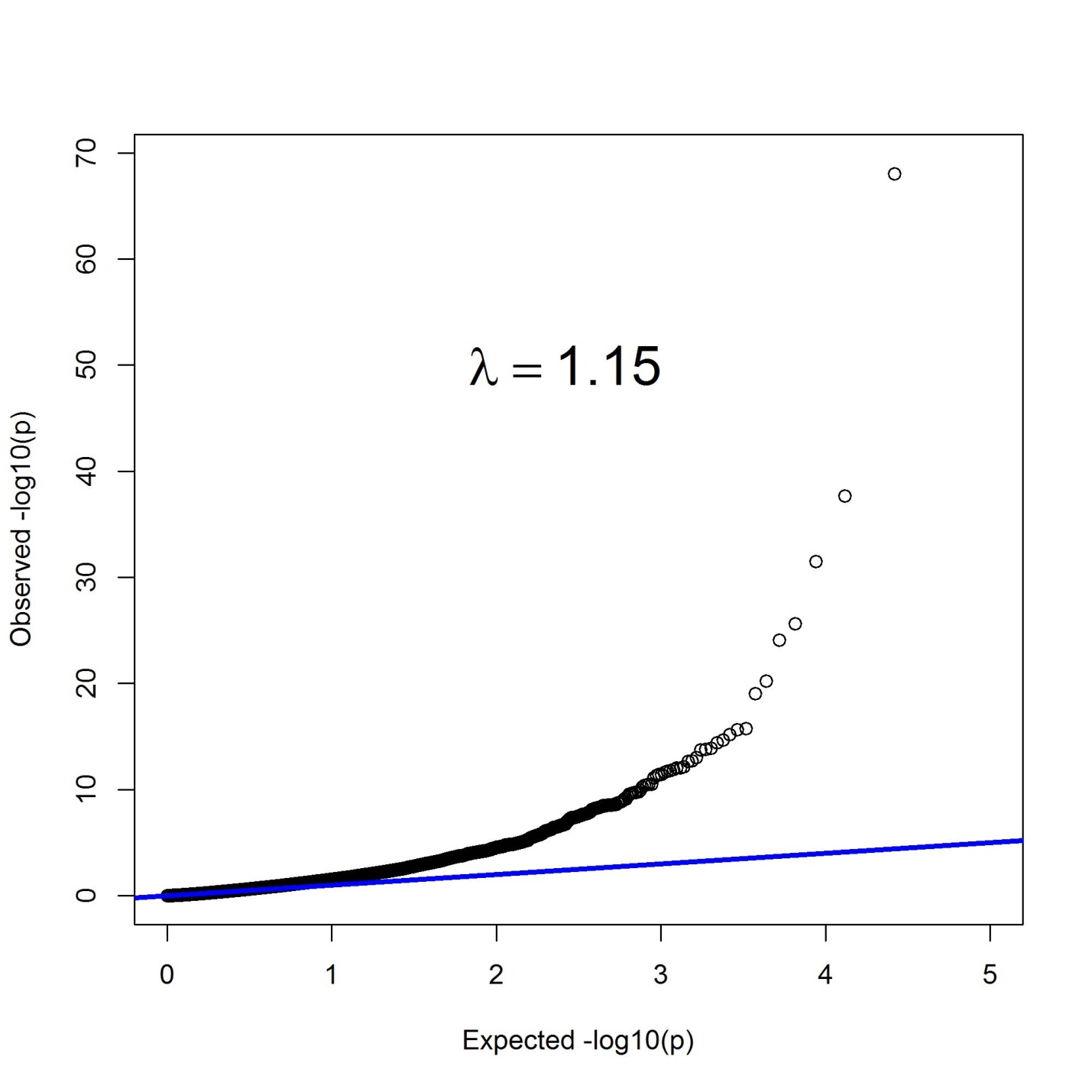


**Supplementary Figure 2. Correlation of z-scores with different lengths of flanking sequences.** Each dot represents one gene. The x-axis represents scores using 50kb flanking sequence, whereas the y-axis represents z-scores using 100kb flanking sequence. They were highly correlated (R^2^=0.88).


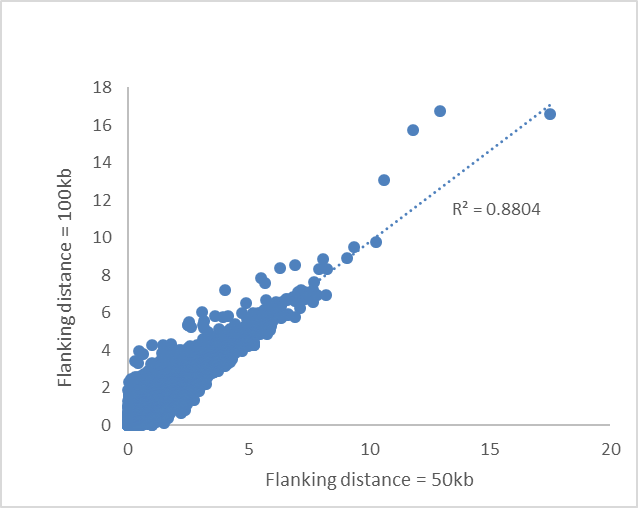


**Supplementary Table 1.** Full list of genes significantly associated with CAD (*P*<0.05/26,228=1.91x10^-6^).

| **Chromosome** | **Gene** | **#SNPs within the gene** | **Association of the gene with CAD (*P* value)** | **Best SNP with the gene** | **Association of best SNP with CAD (*P* value)** |
| --- | --- | --- | --- | --- | --- |
| 9p21.3 | *CDKN2B-AS1* | 283 | 9.45E-69 | rs2891168 | 2.29E-98 |
| 9p21.3 | *CDKN2B* | 96 | 2.04E-38 | rs7028268 | 4.98E-48 |
| 9p21.3 | *CDKN2A* | 119 | 3.42E-32 | rs3217992 | 1.03E-42 |
| 9p21.3 | *CDKN2A-AS1* | 104 | 2.26E-26 | rs3217992 | 1.03E-42 |
| 6q25.3 | *LPA* | 180 | 8.34E-25 | rs55730499 | 5.39E-39 |
| 19p13.2 | *SMARCA4* | 219 | 5.88E-21 | rs56289821 | 4.44E-15 |
| 15q25.1 | *ADAMTS7* | 216 | 9.37E-20 | rs4468572 | 4.44E-16 |
| 19p13.2 | *LDLR* | 256 | 1.71E-16 | rs56289821 | 4.44E-15 |
| 1p13.3 | *SORT1* | 114 | 2.18E-16 | rs7528419 | 1.97E-23 |
| 15q25.1 | *MORF4L1* | 134 | 6.37E-16 | rs4468572 | 4.44E-16 |
| 19p13.2 | *MIR6886* | 182 | 2.13E-15 | rs56289821 | 4.44E-15 |
| 1p13.3 | *MYBPHL* | 113 | 3.96E-15 | rs7528419 | 1.97E-23 |
| 10q11.21 | *C10orf142* | 135 | 1.31E-14 | rs1746050 | 6.28E-13 |
| 6p24.1 | *PHACTR1* | 656 | 1.59E-14 | rs9349379 | 1.81E-42 |
| 1p13.3 | *PSRC1* | 128 | 1.88E-14 | rs7528419 | 1.97E-23 |
| 6q23.2 | *LINC01312* | 113 | 9.18E-14 | rs12202017 | 1.98E-11 |
| 2q33.2 | *WDR12* | 51 | 2.08E-13 | rs115396314 | 5.11E-18 |
| 6q26 | *PLG* | 184 | 2.28E-13 | rs2315065 | 2.88E-34 |
| 1q41 | *MIA3* | 99 | 7.04E-13 | rs67180937 | 1.01E-12 |
| 1q41 | *TAF1A-AS1* | 111 | 9.66E-13 | rs35700460 | 1.38E-12 |
| 1p13.3 | *CELSR2* | 159 | 9.70E-13 | rs7528419 | 1.97E-23 |
| 2q33.2 | *CARF* | 67 | 1.28E-12 | rs115654617 | 3.12E-18 |
| 10q11.21 | *LINC00841* | 222 | 1.57E-12 | rs1870634 | 5.55E-15 |
| 1q41 | *TAF1A* | 132 | 1.96E-12 | rs35700460 | 1.38E-12 |
| 1p32.2 | *PLPP3* | 211 | 2.40E-12 | rs9970807 | 5.00E-14 |
| 15q25.1 | *LOC646938* | 143 | 3.80E-12 | rs4887109 | 2.22E-15 |
| 19q13.32 | *APOC1P1* | 137 | 3.89E-12 | rs4420638 | 7.07E-11 |
| 6q23.2 | *TCF21* | 96 | 4.81E-12 | rs12202017 | 1.98E-11 |
| 1q41 | *AIDA* | 78 | 8.06E-12 | rs67180937 | 1.01E-12 |
| 19q13.32 | *APOC1* | 162 | 3.18E-11 | rs4420638 | 7.07E-11 |
| 19q13.32 | *APOC4* | 108 | 3.30E-11 | rs4420638 | 7.07E-11 |
| 19p13.2 | *CARM1* | 111 | 3.83E-11 | rs12979495 | 4.73E-10 |
| 19p13.2 | *TIMM29* | 88 | 4.24E-11 | rs36045835 | 7.62E-10 |
| 19p13.2 | *YIPF2* | 89 | 5.56E-11 | rs36045835 | 7.62E-10 |
| 10p11.23 | *JCAD* | 269 | 1.21E-10 | rs2487928 | 4.41E-11 |
| 19q13.32 | *APOE* | 168 | 1.64E-10 | rs4420638 | 7.07E-11 |
| 2q33.2 | *NBEAL1* | 84 | 1.82E-10 | rs115654617 | 3.12E-18 |
| 19q13.32 | *APOC4-APOC2* | 113 | 1.84E-10 | rs4420638 | 7.07E-11 |
| 6q25.3 | *SLC22A3* | 181 | 2.10E-10 | rs9457927 | 1.11E-21 |
| 13q34 | *MIR8073* | 261 | 2.37E-10 | rs11838776 | 1.83E-10 |
| 6q25.3 | *LPAL2* | 115 | 2.59E-10 | rs9457927 | 1.11E-21 |
| 1p13.3 | *SARS* | 169 | 4.59E-10 | rs7528419 | 1.97E-23 |
| 1q41 | *BROX* | 86 | 8.00E-10 | rs17465940 | 7.47E-12 |
| 19q13.32 | *TOMM40* | 195 | 8.18E-10 | rs4420638 | 7.07E-11 |
| 2q33.2 | *ICA1L* | 93 | 1.14E-09 | rs115396314 | 5.11E-18 |
| 2q33.2 | *ABI2* | 82 | 1.38E-09 | rs72938351 | 1.85E-14 |
| 19q13.32 | *APOC2* | 104 | 1.66E-09 | rs4420638 | 7.07E-11 |
| 2p11.2 | *VAMP8* | 81 | 1.73E-09 | rs7568458 | 3.62E-10 |
| 19q13.32 | *NECTIN2* | 261 | 2.56E-09 | rs4420638 | 7.07E-11 |
| 2p11.2 | *PARTICL* | 112 | 2.63E-09 | rs7568458 | 3.62E-10 |
| 13q34 | *COL4A1* | 681 | 2.67E-09 | rs4773141 | 2.14E-09 |
| 2q33.2 | *CYP20A1* | 63 | 2.91E-09 | rs72936326 | 4.44E-16 |
| 2p11.2 | *VAMP5* | 83 | 2.94E-09 | rs7568458 | 3.62E-10 |
| 2p11.2 | *TMEM150A* | 73 | 3.03E-09 | rs7568458 | 3.62E-10 |
| 12q24.12 | *ATXN2-AS* | 30 | 3.06E-09 | rs11065979 | 1.93E-10 |
| 2p11.2 | *MAT2A* | 120 | 3.11E-09 | rs7568458 | 3.62E-10 |
| 2p11.2 | *RNF181* | 74 | 3.11E-09 | rs7568458 | 3.62E-10 |
| 2p11.2 | *GGCX* | 122 | 3.15E-09 | rs7568458 | 3.62E-10 |
| 12q21.33 | *ATP2B1-AS1* | 75 | 3.44E-09 | rs111478946 | 1.80E-10 |
| 12q24.12 | *ATXN2* | 54 | 4.00E-09 | rs11065979 | 1.93E-10 |
| 19q13.2 | *CCDC97* | 71 | 4.35E-09 | rs15052 | 2.21E-07 |
| 16q23.1 | *BCAR1* | 276 | 4.41E-09 | rs7188857 | 1.21E-06 |
| 12q24.12 | *BRAP* | 32 | 4.97E-09 | rs11065979 | 1.93E-10 |
| 2q33.2 | *RAPH1* | 84 | 5.49E-09 | rs12693989 | 3.36E-10 |
| 19p13.2 | *C19orf38* | 79 | 5.63E-09 | rs12979495 | 4.73E-10 |
| 19q13.2 | *TGFB1* | 80 | 6.26E-09 | rs15052 | 2.21E-07 |
| 8p21.3 | *LPL* | 225 | 6.83E-09 | rs17411031 | 1.17E-06 |
| 2p11.2 | *C2orf68* | 79 | 7.34E-09 | rs7568458 | 3.62E-10 |
| 1q21.3 | *IL6R* | 157 | 7.86E-09 | rs6689306 | 2.60E-09 |
| 6q25.3 | *SLC22A2* | 178 | 9.51E-09 | rs10080815 | 1.33E-15 |
| 16q23.1 | *LOC100506281* | 246 | 1.32E-08 | rs7188857 | 1.21E-06 |
| 19p13.2 | *SPC24* | 203 | 1.46E-08 | rs17242381 | 1.87E-11 |
| 1q41 | *FAM177B* | 91 | 1.52E-08 | rs71524942 | 3.95E-11 |
| 6q25.3 | *SLC22A1* | 180 | 1.83E-08 | rs9457861 | 4.82E-08 |
| 19q13.2 | *B9D2* | 77 | 1.92E-08 | rs15052 | 2.21E-07 |
| 3q22.3 | *MSL2* | 54 | 1.96E-08 | rs73222236 | 1.41E-06 |
| 16q23.1 | *CTRB1* | 250 | 2.09E-08 | rs7188857 | 1.21E-06 |
| 19p13.2 | *MIR199A1* | 89 | 2.16E-08 | rs12979495 | 4.73E-10 |
| 2q35 | *SNORA115* | 196 | 2.21E-08 | rs2552527 | 1.31E-07 |
| 19p13.2 | *MIR6793* | 83 | 2.88E-08 | rs12979495 | 4.73E-10 |
| 19p13.2 | *TMED1* | 81 | 2.89E-08 | rs12979495 | 4.73E-10 |
| 11p15.4 | *LOC440028* | 122 | 3.12E-08 | rs10840293 | 1.28E-08 |
| 21q22.11 | *LINC00310* | 148 | 3.18E-08 | rs28451064 | 1.33E-15 |
| 12q21.33 | *ATP2B1* | 101 | 3.53E-08 | rs2681472 | 6.17E-11 |
| 2p24.1 | *APOB* | 127 | 3.95E-08 | rs515135 | 3.09E-08 |
| 1q21.3 | *LOC101928101* | 125 | 4.06E-08 | rs6689306 | 2.60E-09 |
| 17p13.3 | *SMG6* | 322 | 4.16E-08 | rs9914266 | 8.92E-08 |
| 1q21.3 | *SHE* | 82 | 4.42E-08 | rs6694817 | 2.96E-09 |
| 17q21.32 | *GIP* | 91 | 4.46E-08 | rs35895680 | 3.76E-07 |
| 17p13.3 | *LOC101927839* | 74 | 4.57E-08 | rs9914266 | 8.92E-08 |
| 17q21.32 | *SNF8* | 84 | 4.62E-08 | rs35895680 | 3.76E-07 |
| 19p13.2 | *DNM2* | 162 | 4.69E-08 | rs12979495 | 4.73E-10 |
| 10q24.32 | *AS3MT* | 93 | 4.94E-08 | rs11191416 | 4.65E-09 |
| 10q24.32 | *BORCS7-ASMT* | 103 | 5.40E-08 | rs11191416 | 4.65E-09 |
| 16q23.1 | *CFDP1* | 232 | 7.06E-08 | rs7188857 | 1.21E-06 |
| 19q13.2 | *TMEM91* | 79 | 7.99E-08 | rs73045269 | 3.41E-07 |
| 10q24.32 | *CYP17A1* | 105 | 9.71E-08 | rs11191416 | 4.65E-09 |
| 10q24.32 | *BORCS7* | 92 | 1.01E-07 | rs11191416 | 4.65E-09 |
| 2p24.1 | *TDRD15* | 74 | 1.25E-07 | rs1712246 | 4.47E-08 |
| 3p21.31 | *AMT* | 35 | 1.81E-07 | rs7623687 | 5.22E-07 |
| 3p21.31 | *TCTA* | 34 | 1.89E-07 | rs7623687 | 5.22E-07 |
| 3p21.31 | *NICN1* | 35 | 1.91E-07 | rs7623687 | 5.22E-07 |
| 6p21.2 | *KCNK5* | 275 | 2.11E-07 | rs56336142 | 1.85E-08 |
| 12q24.12 | *ACAD10* | 43 | 2.21E-07 | rs11065991 | 1.49E-08 |
| 6q23.2 | *TARID* | 445 | 2.28E-07 | rs12202017 | 1.98E-11 |
| 12q21.33 | *POC1B-AS1* | 70 | 2.32E-07 | rs7314459 | 5.80E-09 |
| 17q21.32 | *IGF2BP1* | 148 | 2.36E-07 | rs35895680 | 3.76E-07 |
| 19q13.2 | *HNRNPUL1* | 121 | 2.60E-07 | rs15052 | 2.21E-07 |
| 2p11.2 | *USP39* | 137 | 2.60E-07 | rs7568458 | 3.62E-10 |
| 11p15.2 | *ARNTL* | 216 | 2.68E-07 | rs1351525 | 1.26E-06 |
| 16q23.1 | *CTRB2* | 229 | 3.26E-07 | rs55993634 | 1.66E-06 |
| 3q22.3 | *MRAS* | 108 | 3.32E-07 | rs1199338 | 3.90E-09 |
| 17q21.2 | *ATP6V0A1* | 97 | 3.40E-07 | rs72823056 | 1.50E-06 |
| 3p25.1 | *FGD5* | 266 | 3.50E-07 | rs748431 | 2.14E-07 |
| 12q21.33 | *GALNT4* | 64 | 3.65E-07 | rs7314459 | 5.80E-09 |
| 15q26.1 | *FES* | 185 | 3.66E-07 | rs2521501 | 5.01E-08 |
| 12q21.33 | *POC1B-GALNT4* | 65 | 3.76E-07 | rs7314459 | 5.80E-09 |
| 14q32.2 | *CYP46A1* | 242 | 3.93E-07 | rs10139550 | 1.38E-08 |
| 14q32.2 | *HHIPL1* | 254 | 4.21E-07 | rs10139550 | 1.38E-08 |
| 13q34 | *COL4A2* | 864 | 4.94E-07 | rs11838776 | 1.83E-10 |
| 1q21.3 | *TDRKH* | 130 | 5.48E-07 | rs11810571 | 1.03E-06 |
| 17q21.32 | *UBE2Z* | 89 | 5.65E-07 | rs1962412 | 7.35E-07 |
| 1q21.3 | *TDRKH-AS1* | 113 | 5.72E-07 | rs11810571 | 1.03E-06 |
| 11q22.3 | *MIR4693* | 105 | 5.76E-07 | rs2128739 | 7.05E-11 |
| 2q33.2 | *FAM117B* | 112 | 5.91E-07 | rs72932752 | 1.50E-17 |
| 11p15.4 | *SBF2-AS1* | 176 | 6.45E-07 | rs10840293 | 1.28E-08 |
| 15q26.1 | *FURIN* | 206 | 6.77E-07 | rs2521501 | 5.01E-08 |
| 12q24.12 | *MAPKAPK5-AS1* | 36 | 7.00E-07 | rs11513729 | 6.97E-09 |
| 12q24.12 | *MAPKAPK5* | 51 | 7.24E-07 | rs11513729 | 6.97E-09 |
| 12q24.12 | *ALDH2* | 49 | 7.30E-07 | rs11513729 | 6.97E-09 |
| 3p25.1 | *LINC02011* | 172 | 7.62E-07 | rs13079221 | 5.89E-07 |
| 19q13.32 | *CLPTM1* | 135 | 7.75E-07 | rs4420638 | 7.07E-11 |
| 2q22.3 | *ZEB2-AS1* | 89 | 8.27E-07 | rs17678683 | 3.00E-09 |
| 11p15.4 | *SWAP70* | 190 | 9.94E-07 | rs10840293 | 1.28E-08 |
| 19p13.2 | *KANK2* | 226 | 1.02E-06 | rs4804573 | 3.08E-07 |
| 12q24.12 | *MIR6761* | 36 | 1.06E-06 | rs11513729 | 6.97E-09 |
| 4q32.1 | *GUCY1A1* | 237 | 1.26E-06 | rs72689147 | 6.07E-09 |
| 1q21.3 | *LINGO4* | 127 | 1.34E-06 | rs11810571 | 1.03E-06 |
| 2q22.3 | *LOC105373656* | 91 | 1.35E-06 | rs17678683 | 3.00E-09 |
| 12q21.33 | *POC1B* | 152 | 1.52E-06 | rs7314459 | 5.80E-09 |
| 12q24.13 | *TMEM116* | 51 | 1.59E-06 | rs17696736 | 7.06E-09 |
| 19p13.2 | *ILF3-AS1* | 81 | 1.62E-06 | rs3859514 | 3.35E-06 |
| 3q22.3 | *NME9* | 82 | 1.69E-06 | rs1199338 | 3.90E-09 |

**Supplementary Table 2.** Genes in the CAD-related network.

| ***Gene*** | **Association of the gene with CAD (P value)** | **Number of neighbors** | **Key driver test (P value)** | **Weighted centroid score** |
| --- | --- | --- | --- | --- |
| *UBC* | 1.87E-03 | 22 | 9.29E-07 | 30.16 |
| *CAND1* | 6.91E-01 | 12 | 7.04E-04 | 21.32 |
| *SMARCA4* | 5.88E-21 | 13 | 6.50E-01 | 21.28 |
| *FN1* | 1.74E-04 | 7 | 1.76E-03 | 17.93 |
| *CUL3* | 1.69E-01 | 15 | 2.11E-02 | 17.02 |
| *HDAC2* | 6.72E-02 | 7 | 3.53E-02 | 17.00 |
| *SUMO1* | 1.23E-04 | 11 | 1.77E-02 | 17.00 |
| *APP* | 7.13E-01 | 14 | 1.00E-03 | 16.74 |
| *CDKN2A* | 3.42E-32 | 10 | 8.82E-01 | 15.47 |
| *RNF2* | 8.15E-01 | 12 | 3.51E-02 | 13.37 |
| *CDKN2B* | 2.04E-38 | 1 | 6.98E-01 | 13.34 |
| *CARM1* | 3.83E-11 | 4 | 8.18E-01 | 13.25 |
| *COL4A2* | 4.94E-07 | 2 | 4.16E-02 | 12.70 |
| *ITGA4* | 4.33E-02 | 5 | 3.67E-03 | 12.69 |
| *NPM1* | 6.28E-01 | 12 | 3.61E-02 | 12.59 |
| *STAT3* | 2.20E-03 | 3 | 2.69E-02 | 12.27 |
| *CUL1* | 1.69E-01 | 7 | 1.61E-01 | 12.27 |
| *APOE* | 1.64E-10 | 2 | 5.43E-02 | 12.21 |
| *COL4A1* | 2.67E-09 | 2 | 4.16E-02 | 12.11 |
| *ATXN1* | 8.82E-01 | 6 | 1.11E-03 | 11.04 |
| *RPL6* | 2.53E-06 | 6 | 5.77E-01 | 11.02 |
| *WDR12* | 2.08E-13 | 4 | 2.80E-01 | 10.80 |
| *PHB* | 3.68E-05 | 5 | 8.96E-01 | 10.60 |
| *MORF4L1* | 6.37E-16 | 3 | 4.06E-01 | 10.46 |
| *APOC2* | 1.66E-09 | 1 | 2.72E-01 | 10.33 |
| *FXR2* | 2.86E-02 | 4 | 2.72E-01 | 10.30 |
| *UBE2Z* | 5.65E-07 | 3 | 3.43E-02 | 10.09 |
| *CDK6* | 4.30E-03 | 3 | 1.41E-01 | 9.55 |
| *YWHAZ* | 9.44E-01 | 9 | 7.37E-02 | 9.32 |
| *ABI2* | 1.38E-09 | 3 | 8.94E-03 | 9.21 |
| *TP53* | 7.06E-01 | 6 | 3.67E-02 | 8.91 |
| *PRAM1* | 6.29E-03 | 3 | 1.41E-01 | 8.83 |
| *MDM2* | 4.04E-01 | 5 | 6.19E-02 | 8.79 |
| *RPL13* | 2.07E-05 | 2 | 4.73E-02 | 8.59 |
| *CELSR2* | 9.70E-13 | 2 | 5.30E-01 | 8.53 |
| *TAF1A* | 1.96E-12 | 1 | 3.07E-01 | 8.15 |
| *MIA3* | 7.04E-13 | 1 | 3.91E-01 | 8.13 |
| *DNM2* | 4.69E-08 | 2 | 9.15E-02 | 7.96 |
| *LPL* | 6.83E-09 | 1 | 2.75E-01 | 7.93 |
| *SNF8* | 4.62E-08 | 1 | 2.71E-01 | 7.89 |
| *ATXN2* | 4.00E-09 | 3 | 8.86E-01 | 7.87 |
| *MAPK14* | 2.33E-02 | 4 | 2.03E-01 | 7.83 |
| *USP39* | 2.60E-07 | 3 | 8.86E-01 | 7.82 |
| *SF1* | 5.17E-01 | 6 | 3.73E-02 | 7.75 |
| *VAMP8* | 1.73E-09 | 2 | 1.74E-01 | 7.59 |
| *IGF2BP1* | 2.36E-07 | 3 | 3.36E-01 | 7.40 |
| *BAZ1B* | 1.26E-01 | 3 | 2.69E-02 | 7.35 |
| *PLCG1* | 7.67E-06 | 3 | 1.79E-01 | 7.32 |
| *TMED10* | 2.95E-05 | 2 | 1.00E-01 | 7.18 |
| *CCDC97* | 4.35E-09 | 1 | 3.78E-01 | 7.17 |
| *IKBKE* | 8.29E-01 | 4 | 9.72E-02 | 7.14 |
| *MST1R* | 1.91E-04 | 2 | 3.87E-02 | 7.10 |
| *FURIN* | 6.77E-07 | 2 | 4.73E-02 | 7.09 |
| *CFDP1* | 7.06E-08 | 2 | 7.75E-01 | 7.08 |
| *HNRNPUL1* | 2.60E-07 | 3 | 6.97E-01 | 7.02 |
| *PHF10* | 3.76E-01 | 3 | 7.28E-03 | 7.02 |
| *AIDA* | 8.06E-12 | 1 | 7.29E-01 | 7.01 |
| *YIPF2* | 5.56E-11 | 1 | 5.07E-01 | 7.00 |
| *VAMP5* | 2.94E-09 | 1 | 3.07E-01 | 6.97 |
| *BRAP* | 4.97E-09 | 1 | 6.40E-01 | 6.89 |
| *IL6R* | 7.86E-09 | 1 | 3.09E-01 | 6.89 |
| *MAT2A* | 3.11E-09 | 1 | 3.07E-01 | 6.87 |
| *ILF3* | 3.59E-06 | 6 | 9.17E-01 | 6.83 |
| *SARS* | 4.59E-10 | 2 | 7.36E-01 | 6.78 |
| *VAMP2* | 1.29E-01 | 3 | 1.44E-01 | 6.69 |
| *MYC* | 4.16E-01 | 2 | 3.67E-02 | 6.68 |
| *ACBD3* | 4.49E-01 | 3 | 7.30E-02 | 6.60 |
| *TOMM40* | 8.18E-10 | 1 | 9.36E-01 | 6.35 |
| *AS3MT* | 4.94E-08 | 1 | 3.07E-01 | 6.32 |
| *PCNA* | 9.99E-01 | 6 | 1.21E-01 | 6.26 |
| *SHC1* | 1.08E-05 | 2 | 3.50E-01 | 6.26 |
| *TMED1* | 2.89E-08 | 1 | 4.43E-01 | 6.19 |
| *TRAF1* | 2.07E-01 | 2 | 4.73E-02 | 6.10 |
| *RNF181* | 3.11E-09 | 1 | 8.41E-01 | 6.08 |
| *TMED9* | 5.35E-02 | 3 | 1.51E-01 | 6.05 |
| *CYP20A1* | 2.91E-09 | 1 | 7.29E-01 | 6.05 |
| *KANK2* | 1.02E-06 | 1 | 2.88E-01 | 6.04 |
| *KCNK5* | 2.11E-07 | 1 | 3.07E-01 | 5.97 |
| *BCAR1* | 4.41E-09 | 1 | 3.87E-01 | 5.90 |
| *MSL2* | 1.96E-08 | 2 | 4.32E-01 | 5.89 |
| *ATP6V0A1* | 3.40E-07 | 1 | 5.75E-01 | 5.89 |
| *PPP1R9B* | 2.67E-01 | 3 | 1.47E-01 | 5.78 |
| *SMG6* | 4.16E-08 | 1 | 9.30E-01 | 5.68 |
| *UBE2D3* | 6.24E-01 | 4 | 3.26E-02 | 5.66 |
| *ATP2B1* | 3.53E-08 | 1 | 5.89E-01 | 5.62 |
| *MAPKAPK5* | 7.24E-07 | 1 | 3.78E-01 | 5.62 |
| *FAM117B* | 5.91E-07 | 1 | 5.00E-01 | 5.59 |
| *APOB* | 3.95E-08 | 1 | 6.59E-01 | 5.58 |
| *SREBF1* | 1.37E-05 | 2 | 7.35E-01 | 5.54 |
| *RASA1* | 8.41E-01 | 2 | 3.87E-02 | 5.52 |
| *TCTA* | 1.89E-07 | 1 | 4.88E-01 | 5.28 |
| *RNF5* | 8.83E-02 | 2 | 3.80E-02 | 5.28 |
| *RAD21* | 7.64E-01 | 4 | 2.66E-02 | 5.27 |
| *POC1B* | 1.52E-06 | 1 | 6.39E-01 | 5.25 |
| *FES* | 3.66E-07 | 1 | 8.46E-01 | 5.22 |
| *MCM5* | 9.21E-01 | 2 | 9.15E-02 | 5.21 |
| *DYRK1A* | 8.46E-02 | 3 | 1.56E-01 | 5.08 |
| *ALDH2* | 7.30E-07 | 1 | 7.29E-01 | 5.05 |
| *SWAP70* | 9.94E-07 | 1 | 8.36E-01 | 5.02 |
| *TERF1* | 6.19E-01 | 3 | 7.28E-03 | 4.98 |
| *MAPK10* | 4.87E-01 | 3 | 1.79E-01 | 4.57 |
| *SMG5* | 5.26E-01 | 2 | 3.98E-02 | 4.31 |
